# Statistical model of natural stimuli predicts edge-like pooling of spatial frequency channels in V2

**DOI:** 10.1186/1471-2202-6-12

**Published:** 2005-02-16

**Authors:** Aapo Hyvärinen, Michael Gutmann, Patrik O Hoyer

**Affiliations:** 1 HIIT Basic Research Unit and Dept of Computer Science, University of Helsinki, Finland

## Abstract

**Background:**

It has been shown that the classical receptive fields of simple and complex cells in the primary visual cortex emerge from the statistical properties of natural images by forcing the cell responses to be maximally sparse or independent. We investigate how to learn features beyond the primary visual cortex from the statistical properties of modelled complex-cell outputs. In previous work, we showed that a new model, non-negative sparse coding, led to the emergence of features which code for contours of a given spatial frequency band.

**Results:**

We applied ordinary independent component analysis to modelled outputs of complex cells that span different frequency bands. The analysis led to the emergence of features which pool spatially coherent across-frequency activity in the modelled primary visual cortex. Thus, the statistically optimal way of processing complex-cell outputs abandons separate frequency channels, while preserving and even enhancing orientation tuning and spatial localization. As a technical aside, we found that the non-negativity constraint is not necessary: ordinary independent component analysis produces essentially the same results as our previous work.

**Conclusion:**

We propose that the pooling that emerges allows the features to code for realistic low-level image features related to step edges. Further, the results prove the viability of statistical modelling of natural images as a framework that produces quantitative predictions of visual processing.

## Background

A number of models approach the computational modelling of primary visual cortex by using two processing stages. First, there is a linear filtering with filters that are bandpass, oriented, and spatially localized. In some models, the outputs of the linear filters are half-wave rectified, but this difference is inessential because a rectification is done in the second stage anyway. The second stage then consists of pooling together rectified outputs of the first stage, so that cells that have the same orientation and frequency, as well as similar spatial locations, are pooled together. This pooling is then essentially a summation of rectified outputs of filters of different phases. These two processing steps are assumed to roughly correspond to simple and complex cells in V1, respectively. While there is controversy of the validity of such models, see e.g. [1-3], this is probably the simplest and most succesful approach.

Recent research has seen a number of models that attempt to explain these processing stages based on statistical modelling of natural images (ecologically valid input). First, application of independent component analysis (ICA) [4] or sparse coding [5] shows that the statistically optimal linear features of natural images are very similar to those computed in simple cells in V1 [6-12]. Second, application of a variant of ICA in which some pooling is done in a second stage leads to processing that is similar to what is done in complex cells [13]. Thus, models based on natural image statistics have been able to succesfully reproduce the above-mentioned two stages, and many well-known observations on V1.

It would be most useful if we could use this modelling endeavour in a *predictive *manner, so that we would be able to predict properties of cells in the visual cortex, in cases where the properties have not yet been demonstrated experimentally. This would give testable, quantitative hypotheses that might lead to great advances, especially in the research in extrastriate areas such as V2, whose function is not well understood at this point.

Here, we attempt to accomplish such predictive modelling in order to predict properties of a third processing step, following the two described above. Previously, we have applied a modification of the ICA / sparse coding model on the outputs of modelled complex cells whose input consisted of natural images [14]. The modification consisted of assuming that the coefficients in the generative decomposition, as well as the values of the higher-order features, were all non-negative.

We extend our previous results in two ways. The complex cells in our previous work were all constrained to have the same frequency, which was done in order to reduce the computational load. Here, we first report a technical advance: it is not necessary to make the assumptions of nonnegativity as in [14]. Thus, we are able to use the conventional, computationally optimized ICA algorithms, in particular the FastICA algorithm [15]. We are then easily able to incorporate complex cells of different frequencies in the input without exceeding available computational resources. This enables us to study whether some kind of interaction between different frequencies emerges in the statistically optimal higher-order representation.

## Results

### Experiment 1: Using ordinary ICA with no constraints

As described in Methods, we input a large number of natural image patches into model complex cells that computed the sum of squares of outputs of two simple cells, one odd-symmetric and the other even-symmetric. Then, we performed independent component analysis of the complex cell outputs using the FastICA algorithm.

**Figure 1:**
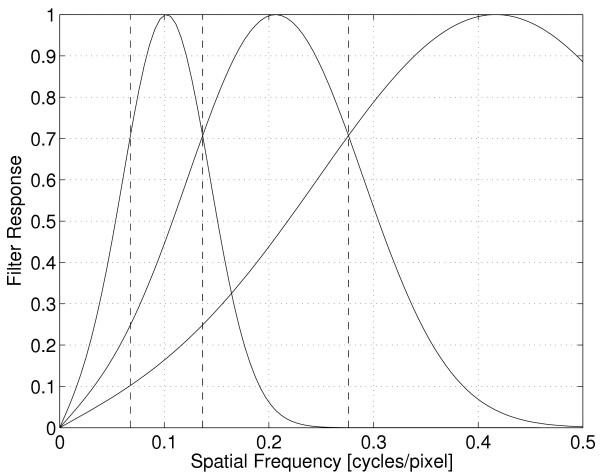
**Frequency tuning of complex cells. **We used three different frequency bands. The underlying Gabor filters had logarithmically spaced frequency peaks and their frequency responses overlapped at the -3dB points. Peak spatial frequencies were chosen as follows: *f*_1 _= 0.1 cycles/pixel, *f*_2 _= 0.21 cycles/pixel and *f*_3 _= 0.42 cycles/pixel. The amplitudes of the Fourier Transforms of the odd-symmetric Gabor filters are shown.

In the first experiment, we used only the output from complex cells in a single frequency band, *f*_2 _in Figure 1. The purpose was to show that the results in [14] can be replicated using ordinary ICA methods.

**Figure 2:**
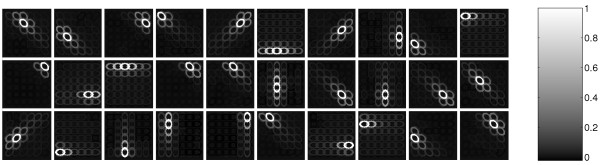
**Basis vectors of Experiment 1. **Random selection of learned basis vectors **a**_*i *_when the complex cells are all in a single frequency band. ICA non-linearity *g *was the tanh nonlinearity *g*_1_. Each patch gives the coefficients of one higher-order feature. Each ellipse means that the complex cell in the corresponding location and orientation is present in the higher-order feature, brightness of ellipse is proportional to coefficient *a*_*ki*_.

The higher-order features are represented by their basis vectors **a**_*i *_which show the contribution of the third-stage feature of index *i *on the activities of complex cells. A collection of the obtained basis vectors is shown in Figure 2 for the nonlinearity *g*_1 _(see Table 1), visualized in the same way as in [14], see Methods. We can see the same kind of emergence of collinear features as in [14]. That is, the higher-order features code for the simultaneous activation of complex cells that together form something similar to a straight line segment.

Those coefficients that are clearly different from zero have almost always the same sign in a single basis vector. Defining the sign as explained in Methods, this means that the coefficients are essentially non-negative. We thus see that the constraint of non-negativity of the basis vectors imposed in [14] has little impact on the results: even without this constraint, the system learns basis vectors which are mainly non-negative.

**Figure 3:**
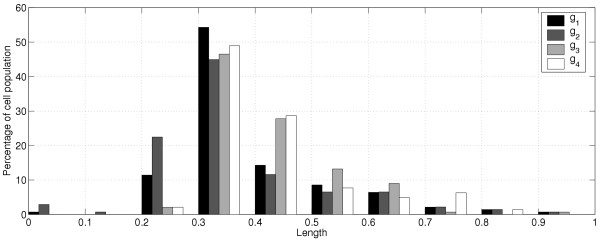
**Comparison of nonlinearities. **Comparison of different FastICA nonlinearities in Experiment 1. The histogram gives the lengths of the contour patterns for the four different nonlinearities *g*_1_,..., *g*_4 _in Table 1.

Other FastICA nonlinearities led to similar basis vectors. However, some led to a larger number of longer contours. Figure 3 shows the distribution of lengths for different nonlinearities. The nonlinearity *g*_4 _(robust skewness) seems to lead to the largest number of long contours.

### Experiment 2: Emergence of pooling over frequencies

In the second experiment, the complex-cell set was expanded to include cells of three different preferred frequencies. In total, there were now 432 complex cells. We performed ICA on the complex-cell outputs when their input consisted of natural images. Thus, we obtained 432 higher-order basis vectors (features) **a**_*i *_with corresponding activities *s*_*i*_.

**Figure 4:**
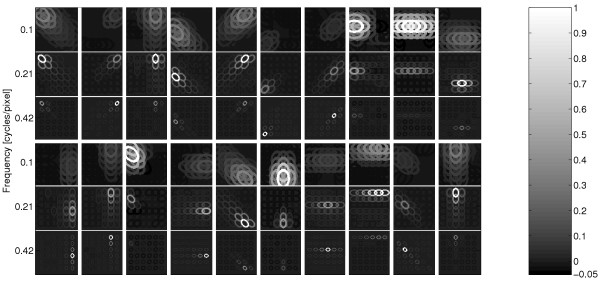
**Basis vectors of Experiment 2. **A random selection of higher-order basis vectors **a**_*i *_estimated from natural images in Experiment 2. ICA nonlinearity *g *was the tanh nonlinearity *g*_1_. Each display of three patches gives the coefficients of one higher-order feature. Each patch gives the coefficients of one higher-order feature in one frequency band. Each ellipse means that the complex cell in the corresponding location, and of the corresponding orientation and frequency is present in the higher-order feature, brightness of ellipse is proportional to coefficient *a*_*ki*_

**Figure 5:**
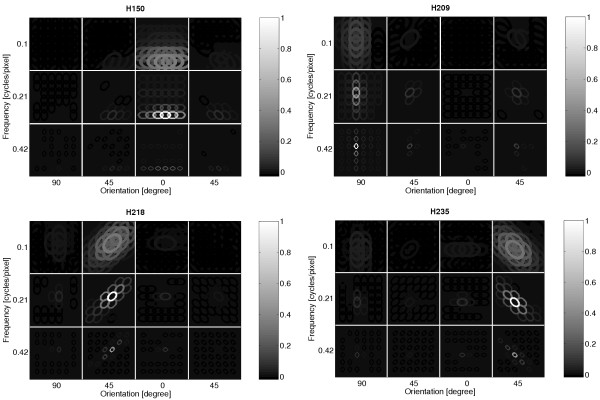
**Basis vectors in detail. **Higher-order basis vectors of four selected higher-order features in Experiment 2, shown in detail. The coefficients in each orientation and frequency band are plotted separately.

We visualized a random selection of higher-order features learned from natural images in Figure 4. The visualization shows that the features tend to be spatially localized and oriented, and show collinearity as in Experiment 1. What is remarkable in these results is that many cells pool responses over different frequencies. The pooling is coherent in the sense that the complex cells that are pooled together have similar locations and orientations. A smaller number of cells is shown in more detail in Figure 5, where the coefficients in all orientation bands are shown separately.

**Figure 6:**
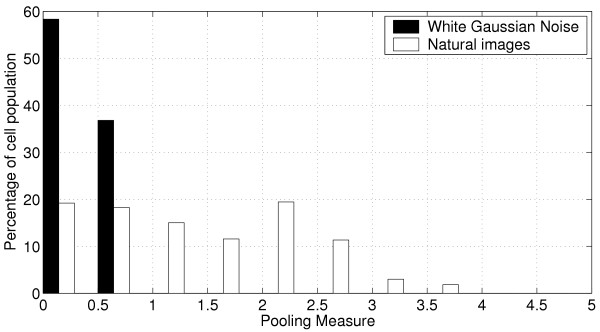
**Pooling measure. **The distributions of the frequency pooling measure in Equation (4) for natural images and white Gaussian noise.

We computed the frequency pooling measure *P*_*i *_in Equation (4) of Methods for the learned basis vectors. The distribution of this measure for natural image input and white Gaussian noise input is shown in Figure 6. The figure shows that frequency pooling according to this measure was essentially nonexistent for white Gaussian noise input, but relatively strong for many basis vectors when the input consisted of natural images. To express this more quantitatively, we computed the 99% quantile for the white Gaussian noise input. Then, 59% of the basis vectors for natural image input had a pooling index *P*_*i *_that was larger than this quantile. (For the 95% quantile the proportion was 63%.) Thus, we can say that more than half of the higher-order basis vectors, when learned from natural images, have a pooling over frequencies that is significantly above chance level.

**Figure 7:**
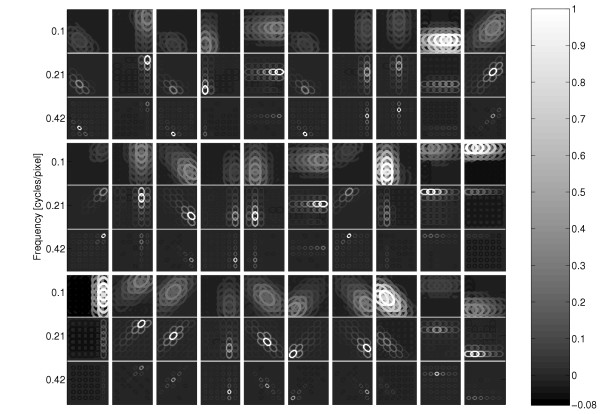
**Basis vectors with significant pooling. **A selection of higher-order basis vectors **a**_*i *_estimated from natural images in Experiment 2. These basis vectors were chosen randomly among those that have frequency pooling significantly above chance level.

To show that the pooling measure is valid, and to further visualize the frequency pooling in the higher-order features, we chose randomly basis vectors learned from natural images that have pooling significantly over chance level (*P*_*i *_above its 99% quantile for white Gaussian noise). These are plotted in Figure 7. Visual inspection shows that in this subset, all basis vectors exhibit pooling over frequencies that respects the orientation tuning and collinearity properties.

**Figure 8:**
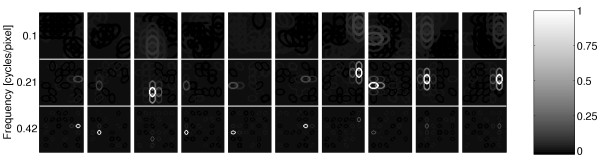
**Basis vectors for white Gaussian noise. **For comparison, higher-order basis vectors estimated from white Gaussian noise, with each frequency band shown separately.

The corresponding results when the input is white Gaussian noise are shown in Figure 8, for a smaller number of higher-order cells. (To make the comparison fair, these were randomly chosen among the 59% that had higher pooling measures, the same percentage as in Figure 7.) Pooling over frequencies as well as collinearity are minimal. Some weak reflections of these properties can be seen, presumably due to the small overlap of the filters in space and frequency, which leads to weak statistical correlations between complex cells that are spatially close to each other or in neighbouring frequency bands.

We also examined quantitatively whether the higher-order features are tuned to orientation. We investigated which complex cell has the maximum weight in **a**_*i *_for each *i *in each frequency band. When the input consisted of natural images, in 86% of the cells the maximally weighted complex cells were found to be located at the hot-spot (*x*_*i*_, *y*_*i*_)* (i.e., point of maximum activity, see Methods for exact definition) and tuned to the preferred orientation of the higher-order feature for *every *frequency *f*. This shows how the higher-order features are largely selective to a single orientation. When the input consisted of Gaussian white noise, only 34% of the cells were found to be orientation-selective according to this criterion.

**Figure 9:**
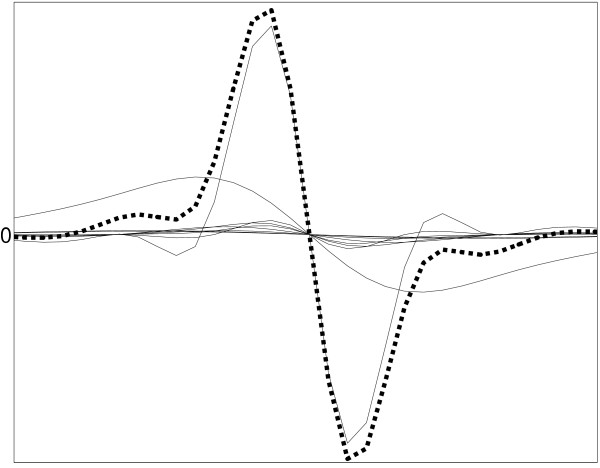
**Image synthesis. **Local image synthesis from the three odd-symmetric Gabor elements that have preferred orientation at the hotspot of a higher-order basis vector (H209 in Figure 5). The thick dotted curve shows the synthesis using coefficients from natural images, and the solid curves show various synthesis results using coefficients learned from white Gaussian noise input.

Finally, we synthesized images from higher-order feature activities to further visualize the higher-order features (see Methods). Figure 9 shows a slice orthogonal to the preferred orientation of one higher-order basis vector (H209 in Figure 5). The intensity of the synthesized image shows no side-lobes (unnecessary oscillations), while representing a sharp, localized edge. In contrast, synthesis in the white Gaussian noise case (also shown in Figure 9) gives curves that have either side-lobes like the underlying Gabor filters, or do not give a sharp localized edge. Thus, the curve obtained from synthesis of the features learned from natural images corresponds better to the notion of an edge. We propose that the utility of pooling over frequencies is due to the broadband nature of real-world edges. Typical edges in natural images are probably not very similar to typical band-pass Gabor functions (or V1 receptive fields) which have oscillations. A proper representation of such broad-band edges would seem to require pooling over different frequencies.

## Discussion

### Frequency channels and edges

What is the functional meaning of the pooling we have found? We propose that this spatially coherent pooling of multiple frequencies leads to representation of an edge that is more realistic than the band-pass edges given by typical Gabor filters [16]. Presumably, this is largely due to the fact that natural images contain many sharp, step-like edges that are not contained in a single frequency band. Thus, representation of such edges is difficult unless information from different frequency bands is combined.

In terms of frequency channels, the model predicts that frequency channels should be pooled together after complex cell processing. Models based on frequency channels and related concepts have been most prominent in image coding literature in recent years, both in biological and computer vision circles. The utility of frequency channels in the initial processing stages is widely acknowledged, and it is not put into question by our results – in fact, the statistical modelling framework does show that using band-pass simple and complex cells is statistically optimal [6,13]. However, the question of when the frequency channels should be pooled or otherwise combined has received little attention [17,18]. Our results point out that a statistically optimal way is to pool them together right after the complex cell "stage", and this pooling should be done among cells of a given orientation which form a local, collinear configuration.

### Related work

Several investigators have looked at the connection between natural image statistics, Gestalt grouping rules, and local interactions in the visual cortex [14,19-21]. However, few has considered the statistical relations between features of different frequencies so far. It should be noted that some related work on interactions of different frequencies does exist in the models of contrast gain control [22].

Compared to our own previous work [14], the main difference seems to be in the frequency tuning of the model complex cells. In [14], the complex cells were all constrained to have the same spatial frequency tuning – just as in Experiment 1 of the present paper. Therefore, it was impossible to obtain results related to frequency pooling. It seems that any differences in the results are not due to differences in the statistical analysis of the complex-cell outputs or the natural image data set used, because in Experiment 1 of the present paper, we essentially replicated the results of [14]. The statistical model for analyzing the outputs of complex cells was somewhat different in our earlier work: the components *s*_*i *_and the coefficients *a*_*ki *_were constrained to be non-negative, following proposals by [23,24]. However, this constraint seems to be immaterial, because even without imposing the constraint, the coefficients turned out to be essentially non-negative (after defining the global sign as described in Methods).

Recent measurements from cat area 18 (somewhat analogous to V2) emphasize responses to "second-order" or "non-Fourier" stimuli, typically sine-wave gratings whose amplitudes are modulated [17,25]. These results and the proposed models are related to our results and predictions, yet fundamentally different. In the model in [25], a higher-order cell pools outputs of complex cells in the same frequency band to find contours that are defined by texture-like cues instead of luminance. The same cell also receives direct input from simple cells of a different frequency, which enables the cell to combine luminance and second-order cues. This is in stark contrast to higher-order cells in our model, which pool outputs of complex cells of different frequencies. They can hardly find contours defined by second-order cues; instead they seem to be good for coding broad-band contours. Furthermore, in [17,25], any collinearity of pooling seems to be absent. This naturally leads to the question: Why are our predictions so different from these results from area 18? We suspect this is because it is customary to think of visual processing in terms of division into frequency channels – "second-order" stimuli are just an extension of this conceptualization. Therefore, not much attempt has been made to find cells that break the division into frequency channels according to our prediction. On the other hand, one can presume that the cells found in area 18 in [17,25] are different from our predictions because they use a different coding strategy from the one used in our model, perhaps related to the temporal aspects of natural image sequences [26,27].

Another closely related line of work is by Zetzsche and coworkers [28,29] who emphasize the importance of decomposing the image information to local phase and amplitude information. The local amplitude is basically given by complex-cell outputs, whereas the physiological coding of the local phases is not known. An important question for future work is how to incorporate phase information in the higher-order units. Some models by Zetzsche et al actually predict some kind of pooling over frequencies, but rather directly after the simple cell stage (see Fig. 16 in [29]).

### Towards predictive modelling

The present results are an instance of predictive modelling, where we attempt to predict properties of cells and cell assemblies that have not yet been observed in experiments. To be precise, the prediction is that in V2 (or some related area) there should be cells whose optimal stimulus is a broad-band edge that has no sidelobes while being relatively sharp, i.e. the optimal stimulus is closer to a step-edge than the band-pass edges that tend to be optimal for V1 simple and complex cells. The optimal stimulus should also be more elongated [30,31] than what is usually observed in V1, while being highly selective for orientation.

Statistical models of natural images offer a framework that lends itself to predictive modelling of the visual cortex. First, they offer a framework where we often see emergence of new kinds of feature detectors – sometimes very different from what was expected when the model was formulated. Second, the framework is highly constrained and data-driven. The rigorous theory of statistical estimation makes it rather difficult to insert the theorist's subjective expectations in the model, and therefore the results are strongly determined by the data. Third, the framework is very constructive. From just a couple of simple theoretical specifications, e.g. non-Gaussianity, natural images lead to the emergence of complex phenomena.

We hope that the present work as well as future results in the same direction will serve as a basis for a new kind of synergy between theoretical and experimental neuroscience.

## Conclusion

We have shown that pooling over complex cells of different frequency preferences emerges when we model the statistical properties of natural images. This is accomplished by applying ordinary ICA on a set of modelled complex cells with multiple frequencies, and inputting natural images to the complex cells. The resulting independent components, as represented by the corresponding basis vectors, code for simultaneous activation of complex cells that have similar orientations, form a collinear configuration, and span multiple frequencies. Thus, statistical modelling of natural stimuli leads to an interesting hypothesis on the existence of a new kind of cells in the visual cortex.

## Methods

### Data and statistical analysis

The natural images were 1008 gray-scale images of size 1024 × 1536 pixels from van Hateren's database, available at  (category "deblurred") [8]. We manually chose natural images in the narrower sense, i.e. only wildlife scenes. From the source images, 50,000 image patches of size 24 × 24 pixels were randomly extracted. The mean grey value of each image patch was subtracted and the pixel values were rescaled to unit variance. The resulting image patch will be denoted by *I*(*x*, *y*).

The complex-cell model was similar to our previous work [14]. The filter bank consisted of a number of complex cells arranged on a 6 × 6 grid. Complex-cell responses *x*_*k *_to natural images were modelled with a classical energy model:


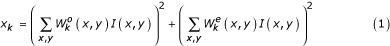


where 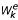 and 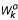 are even- and odd-symmetric Gabor receptive fields whose energies are pooled together in the complex cell. The complex cells had 6 × 6 = 36 different spatial locations, and at each location, four different preferred orientations and three different frequency bands. The aspect ratio was fixed to 1.5 and frequency bandwidth to 1.5 octaves, which implied an orientation bandwidth of 37°, according to the definitions in [8]. The frequency tiling of the Gabor filters is shown in Figure 1, in which all the filters *W *were normalized to unit norm for visualization purposes. The actual normalization we used in the experiments consisted of standardizing the variances of the complex cell outputs so that they were equal to unity for natural image input. The number of complex cells totalled *K *= 36 × 4 × 3 = 432. Note, however, that in Experiment 1 we only used a single frequency band.

Independent component analysis (ICA) was performed on the vector **x **= (*x*_1_,...,*x*_*K*_) using the FastICA algorithm [15]. The orthogonalization approach was symmetric. Different nonlinearities *g *were used, see Table 1. Thus we learned (estimated) a linear decomposition of the form


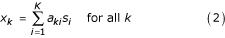


or in vector form


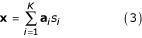


where the vector **a**_*i *_= (*a*_1*i*_,...,*a*_*ki*_) gives a higher-order basis vector. The *s*_*i *_define the values of the higher-order features in the third cortical processing stage.

Note that the signs of the basis vectors are not defined by the ICA model [4], i.e. the model does not distinguish between **a**_*i *_and -**a**_*i *_because any change in sign of the basis vector can be cancelled by changing the sign of *s*_*i *_accordingly. Here, we defined the sign for each vector **a**_*i *_so that the sign of the element with the maximal absolute value was positive.

To obtain a baseline with which to compare our results, and to show which part of the results is due to the statistical properties of natural images instead of some intrinsic properties of our filterbank and analysis methods, we did exactly the same kind of analysis for 24 × 24 image patches that consisted of white Gaussian noise, i.e. the gray-scale value in each pixel was randomly and independently drawn from a Gaussian distribution of zero mean and unit variance. The white Gaussian noise input provides a "chance level" for any quantities computed from the ICA results.

### Analysis of the ICA results

We visualized the resulting higher-order basis vectors **a**_*i *_following [14] by plotting an ellipse at each centrepoint of complex cells. The orientation of the ellipse is the orientation of the complex cell *k*, and the brightness of the ellipse is proportional to the *a*_*ki *_coefficient of the basis vector **a**_*i*_, using a gray-scale coding of coefficient values. In Experiment 1, i.e. the case with a single frequency band, we used this method directly to visualize each higher-order basis vector in a single display. In Experiment 2, i.e. the multifrequency case, we visualized each frequency band separately.

In Experiment 2, we are interested in the frequency pooling of complex cells in different higher-order features. We quantified the pooling over frequencies using a simple measure defined as follows. Let us denote by *a*_*i*_(*x*, *y*, *θ*, *f*_*n*_) the coefficient in the higher-order basis vector **a**_*i *_that corresponds to the complex cell with spatial location (*x*, *y*), orientation *θ *and preferred frequency *f*_*n*_. We computed a quantity which is similar to the sums of correlations of the coefficients over the three frequency bands, but normalized in a slightly different way. This measure *P*_*i *_was defined as follows:


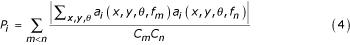


where the normalization constant *C*_*m *_is defined as


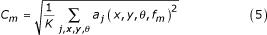


and likewise for *C*_*n*_.

For further analysis of the estimated basis vectors, we defined the preferred orientation of a higher-order feature. First, let us define for a higher-order feature of index *i *the hot-spot (*x*_*i*_, *y*_*i*_)* as the centre location (*x*, *y*) of complex cells where the higher-order component *s*_*i *_generates the maximum amount of activity. That is, we sum the elements of **a**_*i *_that correspond to a single spatial location, and choose the largest sum. This allows us to define the tuning to a given orientation of a higher-order feature *i *by summing over the elements of **a**_*i *_that correspond to the spatial hotspot and a given orientation; the preferred orientation is the orientation for which this sum is maximized. We also computed the length of a higher-order feature as described in [14].

It is also possible to perform an image synthesis from a higher-order basis vector. However, the mapping from image to complex-cell outputs is not one-to-one. This means that the generation of the image is not uniquely defined given the activities of higher-order features alone. A unique definition can be achieved by constraining the phases of the complex cells. We assume that only odd-symmetric Gabor filters are active. Furthermore, we make the simplifying assumptions that the receptive fields *W *in simple cells are equal to the corresponding basis vectors, and that all the elements in the higher-order basis vector are non-negative (or small enough to be ignored). Then, the synthesized image 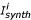 for higher-order basis vector **a**_*i *_is given by


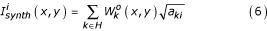


where the square root cancels the squaring operation in the computation of complex-cell responses, and *H *denotes the set of indices that correspond to complex cells of the preferred orientation at the hotspot. Negative values of *a*_*ki *_were set to zero in this synthesis formula.

## Authors' contributions

A.H. conceived the basic idea and the principles of the experimental set-up, and wrote the paper. M.G. performed the experiments and elaborated the experimental set-up. P.O.H. assisted in the experiments and the writing.

## Figures and Tables

**Table 1:** Nonlinearities *g *used in FastICA. The nonlinearities probe the non-Gaussianity of the estimated components in different ways.

*g*_1 _(*y*) = tanh (*y*)	Classic measure of sparseness
*g*_2 _(*y*) = *y *exp(-*y*^2^/2)	More robust variant of *g*_1_
*g*_3 _(*y*) = *y*^2^	Skewness (asymmetry)
*g*_4 _(*y*) = exp(-*y*^2^/2)	Robust variant of *g*_3_
